# Interleukin-23A is associated with tumor growth in *Helicobacter-pylori*-related human gastric cancer

**DOI:** 10.1186/s12935-014-0104-x

**Published:** 2014-10-16

**Authors:** Changming Liu, Ying Zhang, Jie Zhan, Yuanyuan Zhao, Qijun Wan, Huiping Peng, Wei Zhu

**Affiliations:** Department of clinical laboratory, Kunshan Hospital Affiliated to Nanjing University of Chinese Medicine, Kunshan, Jiangsu China; School of Medical Science and Laboratory Medicine, Jiangsu University, Zhenjiang, Jiangsu China

**Keywords:** Interleukin-23A, Interleukin-17A, Nuclear factor-κB, Gastric cancer

## Abstract

**Background:**

Interleukin (IL)-23 is one of the newly identified inflammatory cytokines, and inflammation is also known to be related to the development of gastric cancer (GC). The role of IL-23 in gastric cancer, however, is largely unknown. In the present study, we investigated the expression and possible role of IL-23A in human GC.

**Methods:**

The expression of IL-23A and IL-17A in human GC tissues was determined by immunohistochemistry, and the relationship between IL-23A expression and clinical characteristics of GC was investigated. The serum concentration of IL-23A and IL-17A was also tested by ELISA. The source and role of IL-23A in GC were studied *in vitro* by Flowcytometry, MTS (Owen’s reagent) assay and Western blot.

**Results:**

IL-23A, IL-23 receptor (IL-23R) and IL-17A were all overexpressed in human GC tissues, and the level of IL-23A was well correlated with IL-17A in GC tissues as well as in patient’s serum. Macrophages and GC cells were the main source of IL-23A secretion upon stimulation of *H. pylori* lysate. Furthermore, we found that IL-23A promoted proliferation of GC cell lines via IL-17A/IL-17 receptor antagonist (IL-17RA) /nuclear factor-κB (NF-κB) signaling.

**Conclusions:**

The high expression of IL-23A is associated with GC. IL-23A can promoted GC cells growth by inducing the secretion of IL-17A in tumor microenvironment. Our results suggest that the serum concentration of IL-23A is a good biomarker of poor clinical prognosis in GC patients.

## Background

IL-23A is a subunit of the heterodimeric cytokine, IL-23 by pairing with the other subunit, p40 (IL-12). It is known that IL-23A is expressed and secreted by macrophages and dendritic cells. IL-23 can activate signal transducer and activator of transcription 4 (STAT4) through IL-23R distributed on the membrane of several types of immune cells, including T cells, natural killer (NK) cells, monocytes and dendritic cells. And IL-23 participates immune responses in identifying foreign substances and defending the body against infection and disease [1,2].

IL-23A is involved in the inflammatory response through promoting matrix metalloprotease 9, increasing angiogenesis and reducing CD8^+^ T cells infiltration [3,4]. By working together with IL-6 and transforming growth factor-β1, IL-23 can promote the differentiation of CD4^+^ naive T cells to Th17 cells, which is one of the well accepted T helper cell subsets [5-7]. Th17 cells secrete proinflammatory cytokine IL-17A that can induce Th17 response by stimulating the production of other proinflammatory molecules such as IL-1β, IL-6, tumor necrosis factor-α (TNF-α) and chemokines, resulting in inflammation. It has been reported that IL-17 was closely associated with GC [8,9]. A genome-wide association study has revealed that −197G > A polymorphism at position −197 in the IL-17 promoter region significantly increased GC risk in the general population [10,11], while increased expression of IL-17 was found in patients with GC. IL-17 is also involved in the progression of GC by promoting angiogenesis in the tumor microenvironment [12]. Moreover, persistent inflammation induced in part by IL-17 may contribute to gastric mucosal pathology, thereby increase the risk of GC [9,11,13].

Compared to that of IL-17A, the studies concerning the role of IL-23A in human GC are largely lacking. In the present study, we investigated the expression of IL-23A in GC, and its clinical significance and possible mechanism were involved.

## Results

### IL-23A, IL-23R and IL-17A are excessive in human GC

**Figure 1:**
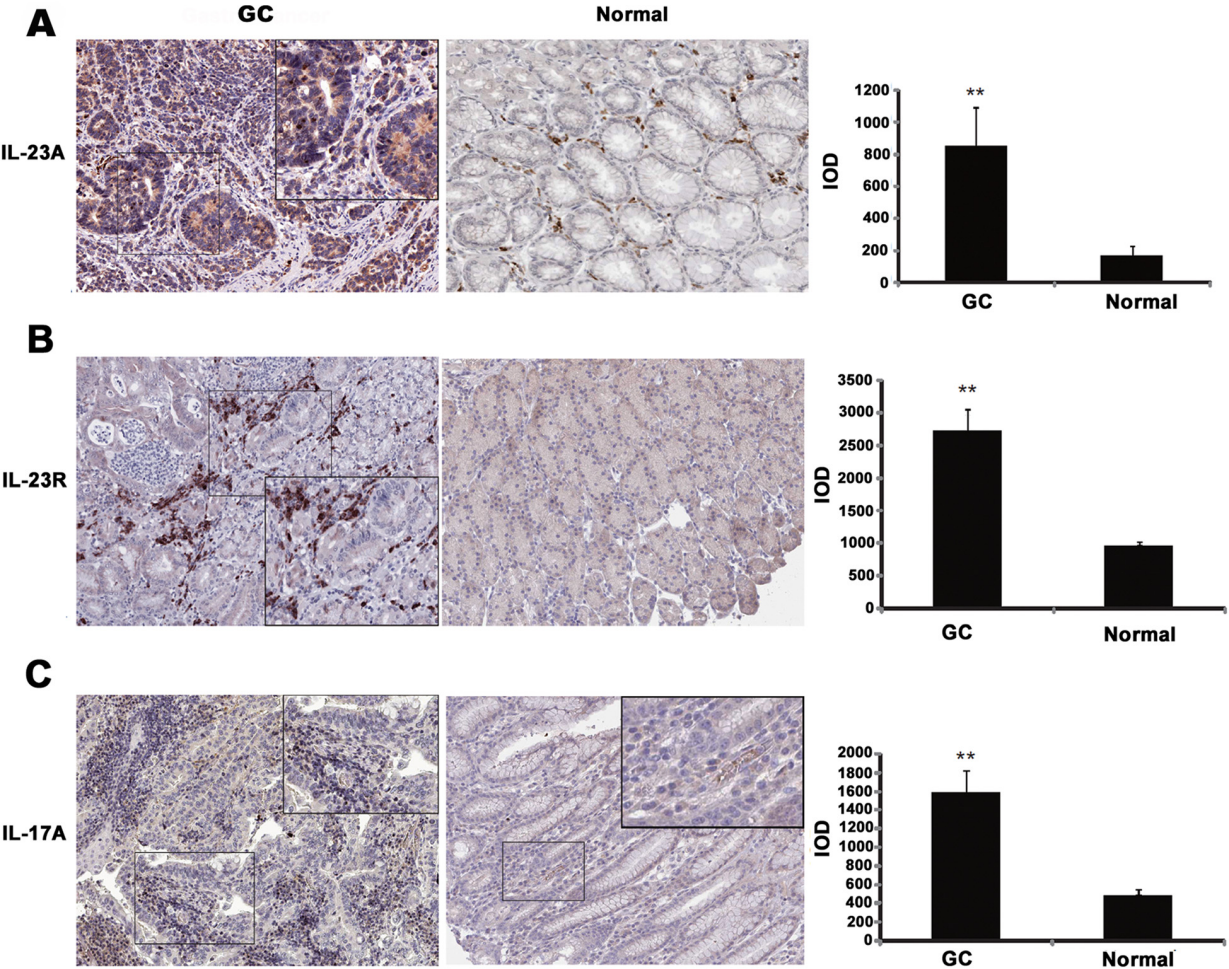
**Expression and distribution of IL-23A, IL-23R and IL-17A in human GC and normal gastric tissue. (A)** Expression and distribution of IL-23A was analyzed by immunohistochemistry in both human GC and normal gastric tissue. Average integrated optical density was obtained by analyzing five fields of view for each slide evaluated by Image-Pro Plus version 5.0. **(B)** Expression, distribution and average integrated optical density of IL-23R. **(C)** Expression, distribution and average integrated optical density of IL-17A. **P < 0.01.

To investigate the expression of IL-23A and the related molecules in human GC, 141 paraffin-embedded tissues were studied for the expression and distribution of IL-23A, IL-23R and IL-17A using immunohistochemistry. First, we observed that IL-23A, IL-23R and IL-17A were all significantly overexpressed in human GC compared to normal controls. Although it was undetectable in the normal gastric glands and slightly positive in infiltrating inflammatory cells, IL-23A was detected at a high level in infiltrating inflammatory cells and cancer cells in cancer tissues (Figure 1A). IL-23R was exclusively expressed in infiltrating inflammatory cells in both cancer and normal tissues, but the expression level and ratio were much higher in cancer tissues (Figure 1B). IL-17A was located mainly in the infiltrated inflammatory cells in GC (Figure 1C). We next investigated the relationship between expression of IL-23A and different clinical characteristics, and found that the level of IL-23A was associated with *H. pylori* infection and tumor burden (Table 1).

**Table 1:** **Clinical characteristics of 141 GC patients**

**Patient demographics**		**IL-23 positive**	**IL-23 negative**	***P*** **value** ^**a**^
Age, y (range)	59 (32–85)	ND	ND	
Sex				0.730
Male	89	45	44	
Female	52	28	24	
Main location				0.613
U	74	36	38	
ML	67	29	38	
Size, cm (range)				0.028^b^
>5	97	74	23	
<5	44	25	19	
Depth of invasion				0.519
T1/T2	88	24	14	
T3/T4	53	29	24	
Lauren classification				0.372
Intestinal	94	45	49	
Diffuse	47	27	20	
Lymph node metastasis				0.401
Positive	69	37	32	
Negative	72	33	39	
*H.pylori* infection				<0.0001^c^
Positive	84	74	10	
Negative	57	28	29	
Stage				0.215
I	32	16	16	
II	45	23	22	
III	43	20	23	
IV	21	10	11	

ND = not determined.

^a^χ^2^ test.

^b^ *P* < 0.05.

^c^ *P* < 0.01.

### IL-23A is correlated with IL-17A secretion in human GC

**Figure 2:**
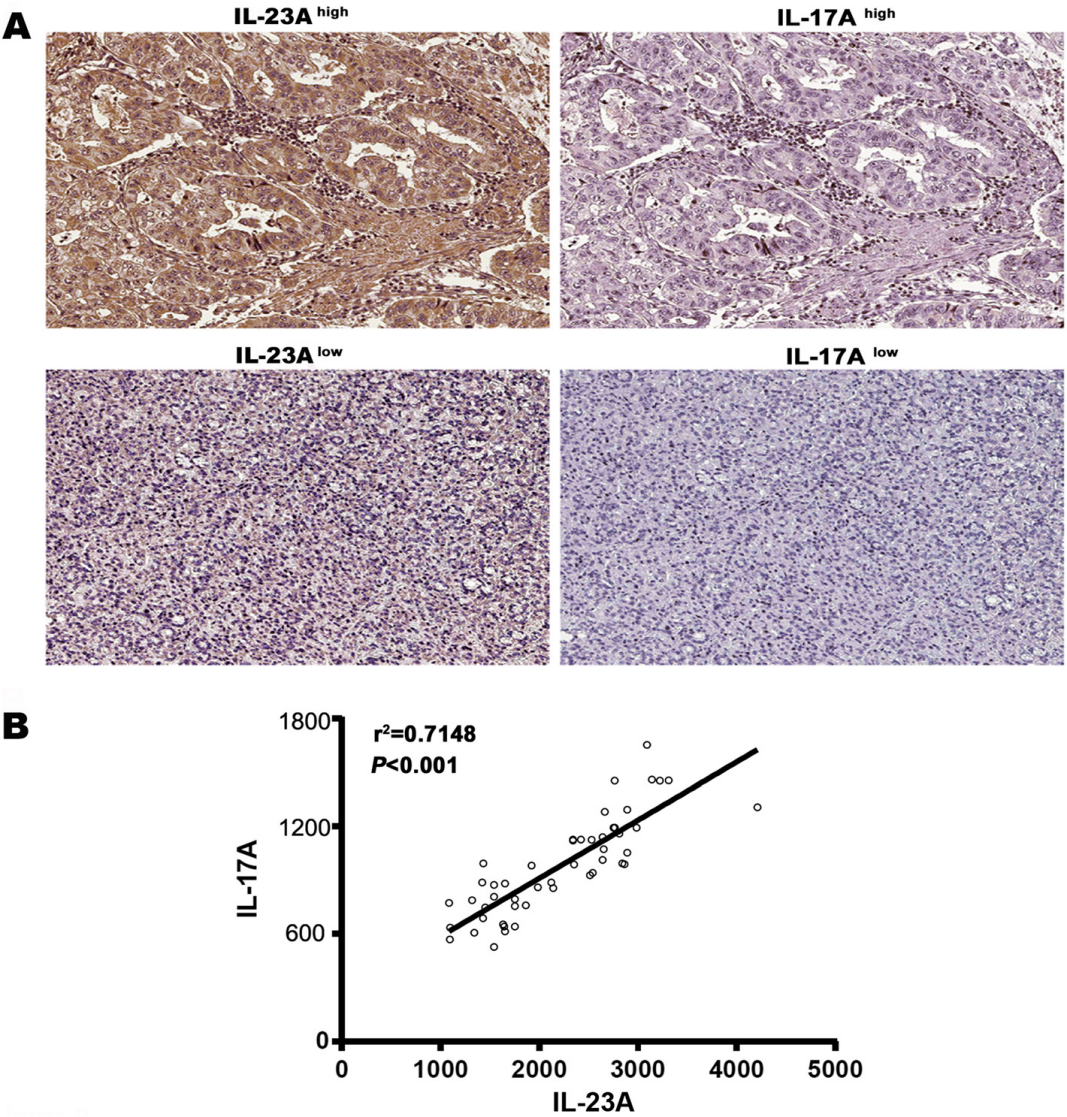
**IL-23A is correlated with IL-17A secretion in human GC. (A)** The correlation between IL-23A and IL-17A in cancer tissues of GC patients was investigated by Immunohistochemistry. **(B)** The correlation between IL-23A and IL-17A within IHC staining of GC patients was accessed by linear correlation test.

Recent studies have shown that secretion of IL-23A was one of the main factors in normal Th17 cell differentiation. We questioned whether IL-23A was also associated with Th17 cell in GC. Indeed, elevated IL-23A expression coincided with increased expression of IL-17A (Figure 2A). To explore the further relationship between them, we quantitated the expression of both cytokines within IHC staining of GC patients and revealed that IL-23A expression was significantly correlated with IL-17A expression through a linear correlation test (r^2^ = 0.7148, *P* < 0.001) (Figure 2B).

### Serum IL-23A concentration is an indicator of poor prognosis in GC patients

**Figure 3:**
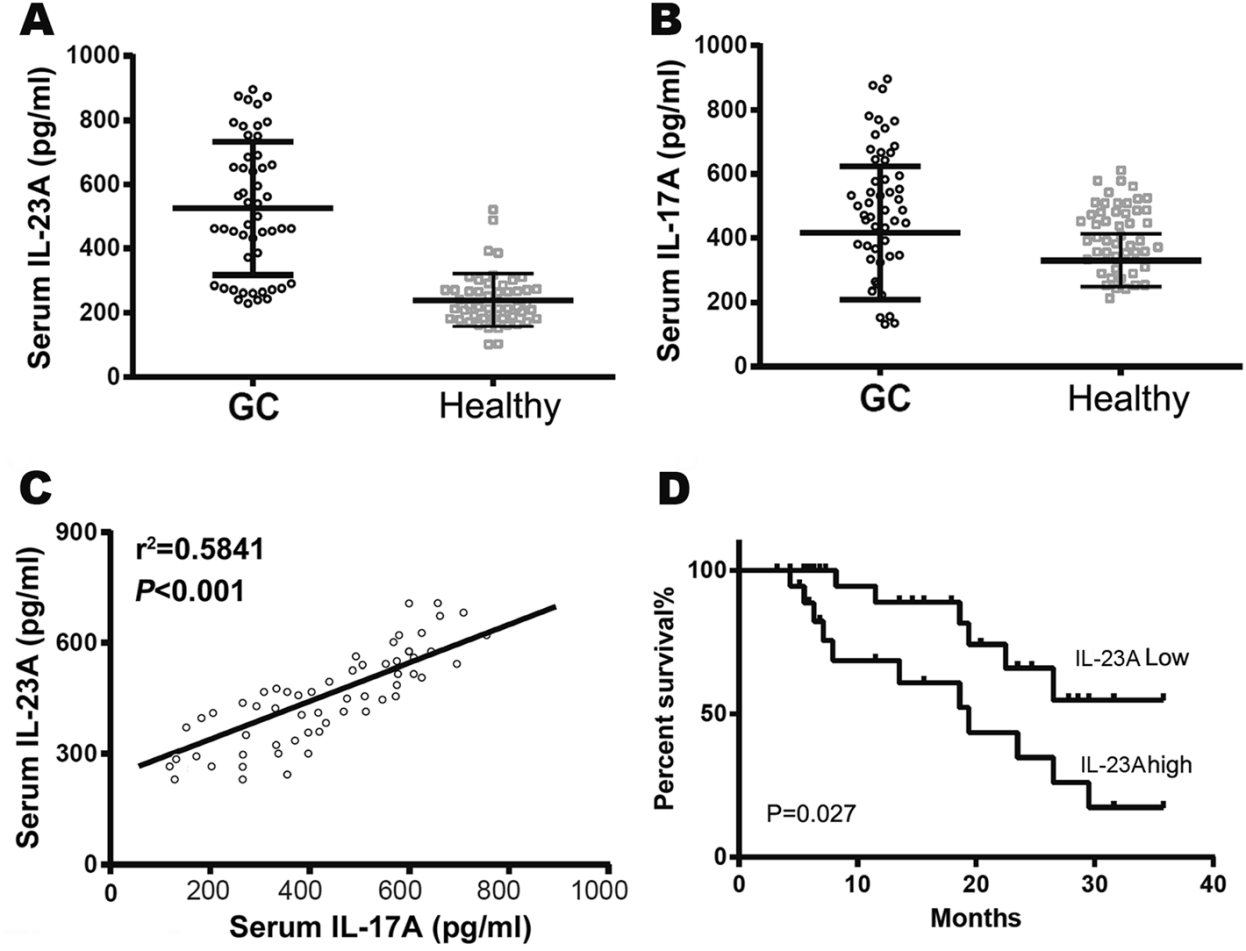
**Serum IL-23A concentration as an indicator of poor prognosis in GC patients. (A)** Serum concentration of IL-23A in GC patients and healthy controls. **(B)** Serum concentration of IL-17A in GC patients and healthy controls. **(C)** The correlation between IL-23A and IL-17A in serum of GC patients was accessed by linear correlation test. **(D)** The Kaplan–Meier curves for OS of GC patients with different expression of IL-23A.

Both IL-23A and IL-17A may be secreted into the blood, therefore, we hypothesized that the level of IL-23A and IL-17A in the serum of GC patients or healthy controls may be determined by ELISA as biomarkers. We first detected the level of both cytokines as 213 ± 75 pg/ml for IL-23A and 286 ± 101 pg/ml for IL-17A in 50 healthy individuals (Figure 3A and B). The serum level of both cytokines were increased significantly in GC patients as 517 ± 247 pg/ml for IL-23A and 422 ± 284 pg/ml for IL-17A (Figure 3A and B). And the linear correlation of serum concentration between IL-23A and IL-17A was also seen in GC patients (r^2^ = 0.5841, *P* < 0.001) (Figure 3C). The threshold of healthy controls was obtained according to the 95% confidence interval (CI), which was 285 pg/ml for IL-23A. According to the threshold, the GC patients were divided into IL-23A high and IL-23A low subsets. To compare their clinical prognosis between the two subsets of GC patients, serum IL-23A expression was examined using the Kaplan–Meier method. We found that IL-23A level (>285 pg/ml) was significantly associated with shorter overall survival (OS) [*P* = 0.027, hazard ratio (HR) 2.246, 95% CI: 1.212–4.160] (Figure 3D).

### IL-23A can be secreted by macrophages and GC cells

**Figure 4:**
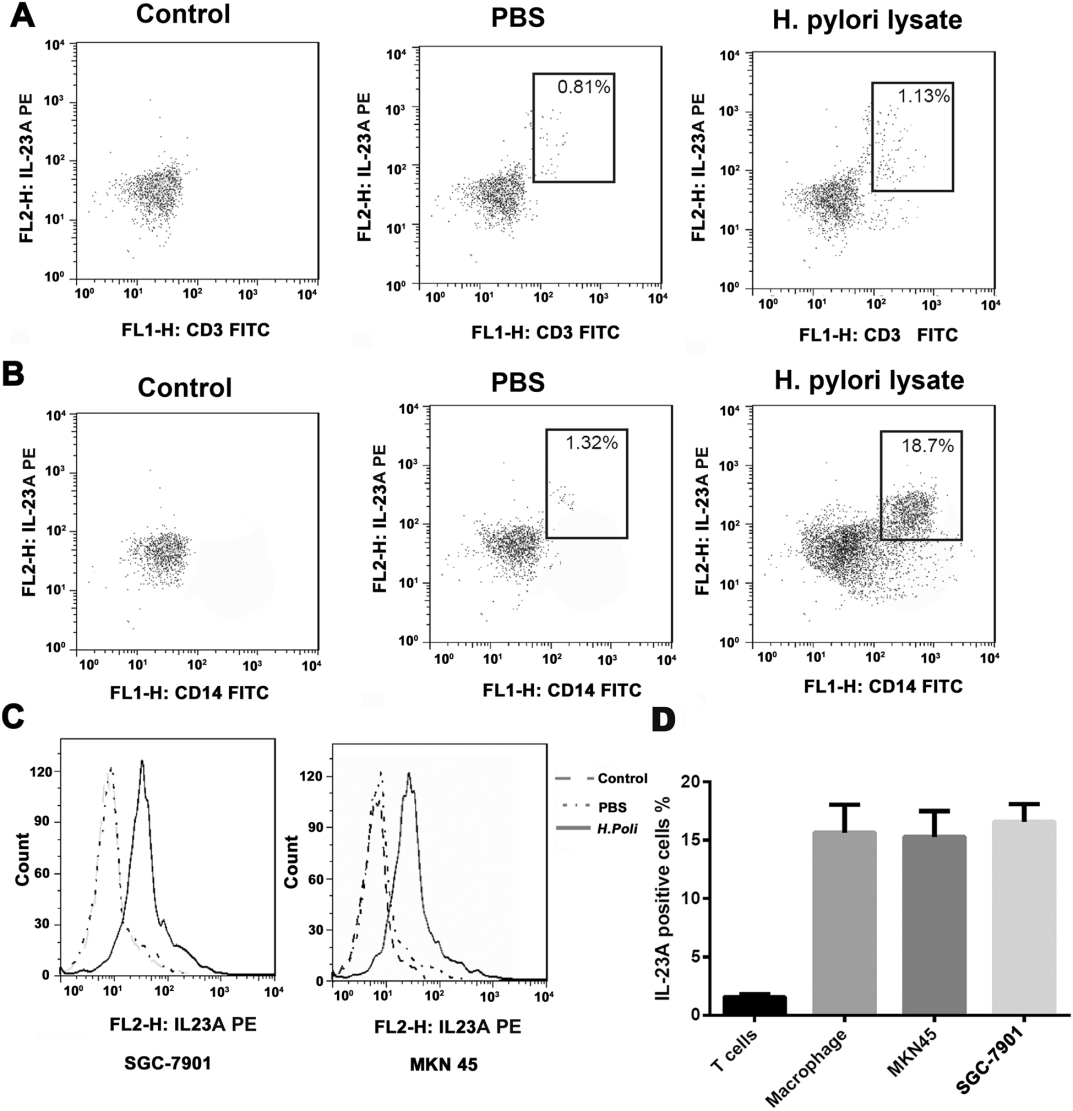
**IL-23A is secreted by macrophages and GC cells. (A)** The expression of IL-23A and cell surface marker CD3 were detected by Flowcytometry in T cells with different treatment. **(B)** The expression of IL-23A and cell surface marker CD14 were detected by Flowcytometry in macrophages with different treatment. **(C)** The expression of IL-23A was detected by FCS in SGC-7901 and MKN45 cells treated with *H. pylori* lysate. **(D)** The ratio of IL-23A positive cells in T cells, macrophages and GC cell lines.

Immunohistochemical staining showed that IL-23A was abundantly expressed in multiple cell types, including T cells, macrophages and GC cells (Figure 1A). To examine the exact origin of IL-23A, we accessed IL-23A expression in an *vitro* stimulation system using *H. pylori* lysate as the cytokine-inducing agent, which based on a previous observation that *H. pylori* was a robust inducer for the secretion of IL-23A. In T cells, IL-23A secretion was increased slightly upon stimulation with *H. pylori* lysate (Figure 4A). In macrophages, the number of IL-23A-positive cells increased from 1.15 ± 0.18% to 13.21 ± 6.21% (Figure 4B). While in GC cell lines, IL-23A-positive SGC-7901 cells increased from 2.64 ± 1.12% to 13.11 ± 3.12%, and IL-23A positive MKN45 cells increased from 1.16 ± 0.46% to 17.55 ± 5.42% (Figure 4C). Overall, macrophages and GC cells showed *H. pylori* lysate-induced stimulation of IL-23A secretion (Figure 4D).

### IL-23A promotes survival of GC cells through IL-17A/IL-17RA/nuclear factor (NF)-κB signaling

**Figure 5:**
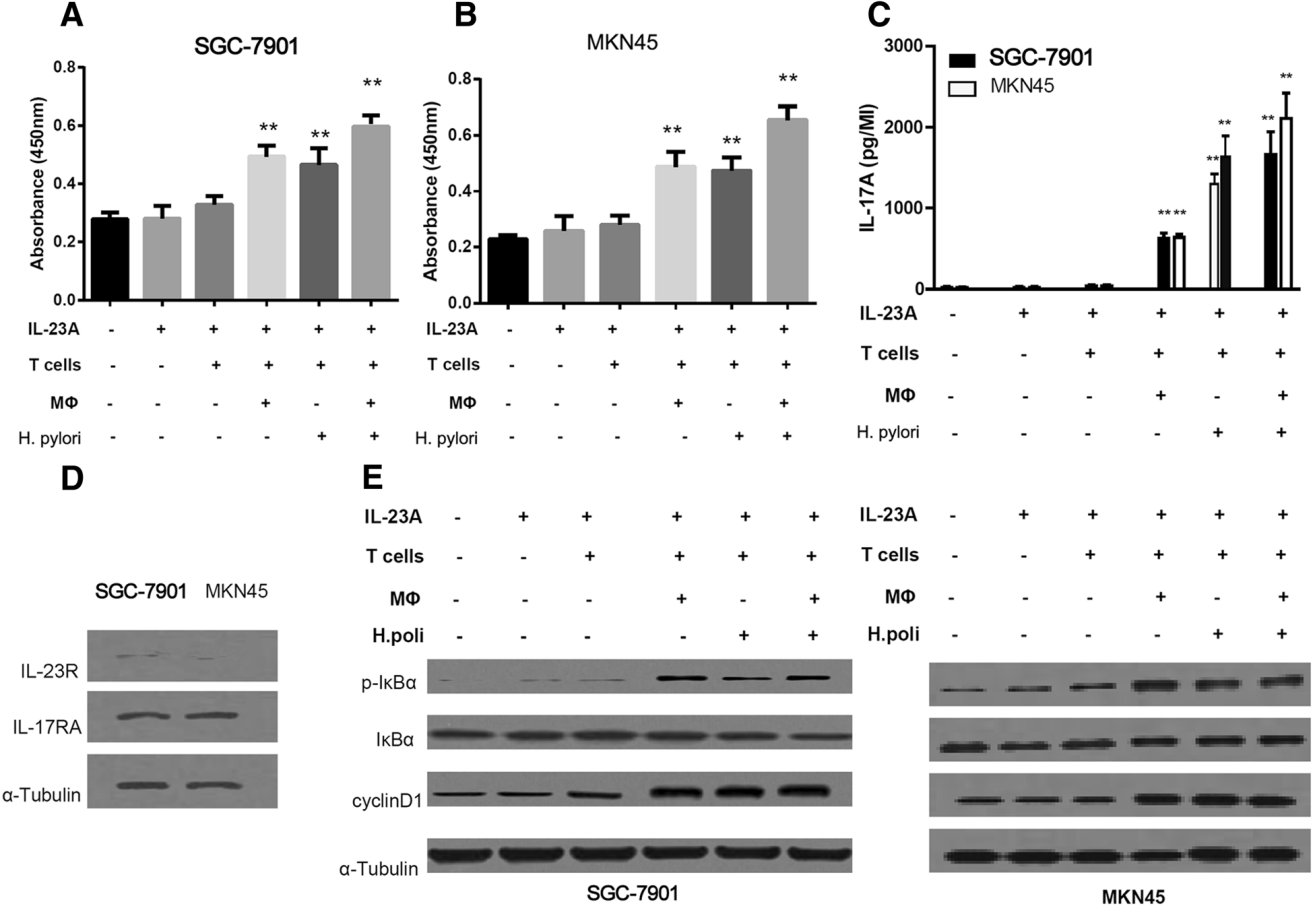
**IL-23A promotes survival of GC cell lines through IL-17A/IL-17RA/NF-**κ**B signaling. (A)** The cell viability of SGC-7901 with the treatment was examined. **(B)** The cell viability of MKN45 with the treatment was examined. **(C)** The concentration of IL-17A in the cell culture medium of SGC-7901 and MKN45 were determined by ELISA. **P < 0.01. **(D)** The expression of IL-17RA and IL-23R in both MKN45 and SGC-7901. **(E)** The expression of p-IkBa, IkBa and CyclinD1 in both MKN45 and SGC-7901.

To investigate the effect of IL-23A on tumor growth, a co-culture assay *in vitro* was utilized. First, we found that IL-23A had no significant effect on cell proliferation when the GC lines SGC-7901 and MKN45 were treated with human recombinant IL-23A directly. We next found that there was no significant effect on tumor cell SGC-7901 or MKN45 growth co-cultured with naive T lymphocytes in the presence of IL-23A. However, the significant cell-growth-promoting effect was seen when either macrophages or *H. pylori* lysate was added to the co-culture system. When both macrophages and *H. pylori* were added, the effect was synergistic (Figure 5A and B).

We investigated the possible mechanism underpinning the association between IL-17A and IL-23A. Concentration of IL-17A in the culture medium was determined, and there was no significant difference when GC cells were treated with IL-23A alone or IL-23A plus T lymphocytes. However, the secretion of IL-17A increased when either *H. pylori* lysate or macrophages was added (Figure 5C). Activation of NF-κB signaling was also examined, and the expression of both IL-17RA and IL-23R was detected in both SGC-7901 and MKN45 cells, relatively strong expression of IL-17RA and almost no expression of IL-23R were identified (Figure 5D). Phosphorylated IκBα and cyclinD1, the products of NF-κB signaling, were both increased in SGC-7901 and MKN45 cells along with increased presence of IL-17A (Figure 5E). IL-23A was secreted from both macrophages and GC cells and promoted cancer proliferation through IL-17A/IL-17RA/NF-κB signaling.

## Discussion

GC is the fourth most common cancer and the second leading cause of cancer-related death worldwide. Among several histological types, intestinal-type GC is commonly associated with *H.Pylori* infection, and its course is characterized by acute gastritis, chronic gastritis, gastric atrophy, intestinal metaplasia, and finally, formation of GC [10].

The exact causes of GC are unknown, but a number of factors can increase the risk of the disease, including sex, race, genetics, geography, blood type, advanced age, family history, and *H.pylori* infection of the stomach. *H. pylori* infects the lining of the stomach and causes chronic inflammation and ulcers. The detailed molecular pathways responsible for the stimulation of *H. pylori*are still not completely known [14-16].

Several studies have reported an immune effector molecules of *H. pylori*, such as CagA. CagA promoted expression of the proinflammatory chemokines IL-8, CXC-chemokine ligand 2 (also known as macrophage inflammatory protein) and the antimicrobial peptide human β-defensin-2 through activation of NF-κB signaling [17]. Other studies have revealed that CagA translocated into epithelial cells by inducing high levels of IL-8 infected by certain *H. pylori* strains, accompanied by induction of an inflammatory response [16,18,19]. Ras-dependent kinases, extracellular signal-regulated kinase (ERK)1 and ERK2, are also activated by CagA-triggered tyrosine phosphorylation, leading to activation of the transcription factors NF-κB, activator protein-1, and ultimately, IL-8 production by host cells [9].

The mechanism linked Hp infection and Th17 response was not fully revealed. Some of the cytokines such as IL-1β and IL-21 were reported to be associated. Lee et al. revealed that persistent Hp infection can induce Hp-specific Th17 cells, rather than other immune cells that have also been described to secrete IL-17A. Moreover, IL-1β was also persistently elevated, and neutralization of IL-1β reduced the Hp-specific IL-17A response, suggesting a functional association between IL-1β and the persistent Th17 response [20]. Other group also revealed that IL-21 regulates Th1 and Th17 effector responses during chronic H. pylori infection in a STAT1- and STAT3-dependent manner, therefore playing a major role controlling H. pylori infection and gastritis [21].

We reported that IL-23A was secreted from GC cells and macrophages. We found that serum level of IL-23A was an indicator of poor prognosis in GC patients, and its expression was related to tumor volume and *H.pylori* infection. Our results also indicated that IL-23A had no direct effect on the proliferation of cancer cells. However, it did affect the tumor microenvironment by increasing secretion of IL-17A, which is a hallmark cytokine of Th17 cells. In conclusion, we found that IL-23A could be a potential index and target for diagnosis and treatment of GC.

## Conclusions

In conclusion, this study showed that the high expression of IL-23A is associated with GC. IL-23A may induce the secretion of IL-17A activate IL-17A/IL-17RA/NF-κB signaling in tumor microenvironment. Serum IL-23A concentration is an indicator of poor prognosis in GC patients and IL-23A will be a potential index and target for diagnosis and treatment of GC.

## Materials and methods

### Patients

The present study included 141 patients with GC who underwent surgery from 2008 to 2013 at the Kunshan People’s Hospital, Kunshan, Jiangsu and Kunshan Hospital Affiliated to Nanjing University of Chinese Medicine. Documented informed consent for gene expression analyses of all tissues was obtained from all patients prior to surgery. This study was approved by the Local Ethics Committee of Kunshan People’s Hospital. Histological subtype according to Lauren’s classification [22] was determined after a review of tumor sections by two pathologists. Follow-up data were available from all GC patients, who were assessed at 3, 6,12 months and then every 6 months for 5 years or until death.

### Immunohistochemistry

All tissues were removed and fixed in 4% paraformaldehyde overnight at 4°C, then processed and sectioned at 5 μm thickness. Sectioned slides were stained by immunohistochemistry for IL-23A, IL-23R and IL-17A (Santa Cruz Biotechnology), followed by incubation with secondary antibody at 37°C for 30 min and reaction with DAB reagent for 5–10 min. The slides were mounted with neutral gum for microscopic examination. Cells with brown intracellular granules (cytoplasm or nucleus) were considered to be positively stained.

### ELISA

Concentration of IL-23A and IL-17A in serum of GC patients and healthy candidates were measured using commercially available sandwich ELISA kits (eBioscience).

### Stimulation of IL-23A *in vitro*

T cells and macrophage was isolated from peripheral blood mononuclear cells by using isolation kit to T cells and monocyte respectively (Miltenyi Biotec). T cells and macrophages plus GC cell lines (MKN45 and SGC-7901 were purchased from ATCC) were maintained *in vitro* and stimulated by *Helicobacter pylori* lysate (NCTC 11637, CagA + and VacA+) and analyzed by intracellular cytokine staining. For intracellular cytokine staining, T cells were stimulated at 37°C for 5 hours with a Leukocyte Activation Cocktail (BD Pharmingen). Furthermore, T cells, macrophages and GC cell lines were stained with surface markers, fixed, and permeabilized with IntraPre Reagent (Beckman Coulter), and finally stained with intracellular markers. Data were acquired on FACSVantage SE and analyzed with CellQuest software. Fluorochrome-conjugated mAbs against IL-23A were purchased from BD Pharmingen (Cat. 562468).

### Flowcytometry

For intracellular cytokine staining, cells were stimulated at 37°C for 5 hours with a Leukocyte Activation Cocktail (BD Pharmingen). Cells were then stained with surface markers, fixed, and permeabilized with IntraPre Reagent (Beckman Coulter), and finally stained with intracellular markers. Data were acquired on FACSVantage SE and analyzed with CellQuest software. Fluorochrome-conjugated monoclonal antibodies (mAbs) against IL-23A, CD3 served as marker for T cells and CD14 serve as markers for monocyte and macrophage were purchased from BD Pharmingen.

### MTS assay

Cultured cells were plated at a density of 6 × 10^3^ cells/well in a 96-well plate and maintained with DMEM plus 10% fetal calf serum (Invitrogen). Cell viability was evaluated by MTS assay. CellTiter 96Aqueous One Solution Reagent (Promega) was added to each well according to the manufacturer’s instructions, and the plates were returned to the incubator. After 4 hours, cell viability was determined by measuring the absorbance at 490 nm using a computer controlled plate-reader.

### Western blot

Proteins were extracted from cells and quantitated using a protein assay (Bio-Rad Laboratories, Hercules, CA). Protein samples (30 μg) were fractionated by SDS-PAGE and transferred to a nitrocellulose membrane. Immunoblot was carried out using antibodies against p-IκBα, IκBα, CyclinD1 and IL-17RA (Santa Cruz Biotechnology). The results were visualized using a chemiluminescent detection system (Pierce ECL substrate western blot detection system; Thermo Scientific) and exposed to autoradiography film (Kodak XAR film).

### Statistical analysis

The results are expressed as mean ± SD of at least triplicate experiments. Comparisons between two groups were performed using the Mann–Whitney *U* test. The comparison of IL-23A expression between various clinical characteristics was analyzed using the χ^2^ test. Correlation analysis between expression of IL-23A and IL-17A in GC tissues was performed using Spearman non-parametric relation analysis. Survival curves were estimated using the Kaplan–Meier product-limit method, and significant differences between the survival curves were determined using the log-rank test. *P* < 0.05 was considered statistically significant.

## Abbreviations

IL-23: Interleukin-23
GC: Gastric cancer
IL-23R: IL-23 receptor
IL-17RA: IL-17 receptor subunit A
NF-κB: Nuclear factor-κB
STAT4: Signal transducer and activator of transcription 4
NK: Natural killer
